# The influence of network structure on neuronal network dynamics

**DOI:** 10.1186/1471-2202-13-S1-P79

**Published:** 2012-07-16

**Authors:** Duane Q Nykamp

**Affiliations:** 1 School of Mathematics, University of Minnesota, Minneapolis, MN USA 55455

## 

We investigate the influence of network structure on the dynamics of neuronal networks, with a focus on the emergence of synchronous oscillations. Network structure is specified using the framework of second order networks, a network model that captures second order statistics (correlations) among the connections between neurons. We demonstrate that the frequency of a chain motif in the network plays a crucial role in influencing network dynamics, not only modulating the emergence of synchrony but also possibly increasing the range of possible network behaviors.

**Figure 1:**
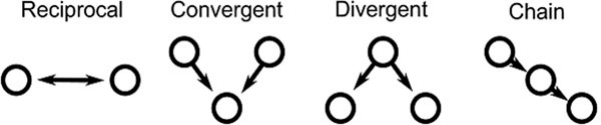
The four second order edge motifs of reciprocal, convergent, divergent, and causal connections. The frequency of these motifs determine the second order statistics, or correlations, among the network connections.

